# ERp29 inhibits tumorigenicity by suppressing epithelial mesenchymal transition in gastric cancer

**DOI:** 10.18632/oncotarget.20225

**Published:** 2017-08-12

**Authors:** Jing Wu, Yuanyan Yang, Shenshen Gao, Hong Jiang, Xin-Qiong Wang, Yuan Xiao, Xue-Hua Chen, Pu Li, Chun-Di Xu

**Affiliations:** ^1^ Department of Pediatrics, Ruijin Hospital and Ruijin Hospital North, Shanghai Jiao Tong University School of Medicine, Shanghai, People’s Republic of China; ^2^ Department of Oncology, East Hospital, Tongji University School of Medicine, Shanghai, People’s Republic of China; ^3^ Department of Human Resource, Ruijin Hospital North, Shanghai Jiao Tong University School of Medicine, Shanghai, People’s Republic of China

**Keywords:** ERp29, EMT, ERK1/2, AKT, gastric cancer

## Abstract

ERp29 is a novel endoplasmic reticulum (ER) protein that plays an important role in protein unfolding and secretion. Recently, it has been reported to be widely implicated in control of tumorigenesis in some tumors. However, the potential function of ERp29 in gastric cancer remains poorly understood. In this study, we found that the positive rate of ERp29 in gastric cancer tissues was significantly lower than that in adjacent non-tumor tissues. And tumor with high ERp29 expression had inclinations towards smaller tumor size and earlier TNM stage. The *in vitro* experiments indicated that over-expression of ERp29 in gastric cancer cells significantly suppressed the proliferation and migration of tumor cells, which is consistent with the result of the *in vivo* animal experiments. Furthermore, our mechanistic investigations revealed that ERp29 reversed EMT process in gastric carcinoma, and its effect was related to the inactivation of ERK1/2 and AKT phosphorylation. Thus, we conclude that ERp29 acts as a tumor suppressor gene in gastric cancer, and is expected to become a novel target of the treatment of GC.

## INTRODUCTION

Gastric cancer is one of the most common malignant tumors and causes a high hazard to human health [1-3]. Despite its decline in incidence in developed countries, gastric cancer remains the second leading cause of cancer-related death in worldwide. In China, about 400,000 patients were newly diagnosed with gastric cancer and approximately 300,000 died of the disease each year. In spite of the development in surgery, chemotherapy and molecular targeted therapies, the prognosis of advanced gastric cancer remains dismal [4]. Additionally, potential biomarkers for early diagnosis and therapeutic targets for GC are still limited. Therefore, there is an urgent need to identify valuable diagnostic markers and targets for treatment of GC.

ERp29 is a novel endoplasmic reticulum (ER) protein that plays an important role in protein unfolding and secretion [5]. Recently, abnormal expression of ERp29 was found in many kinds of malignant tumors, such as basal cell carcinoma [6], lung cancer [7], breast cancer [8] and colorectal cancer [9, 10]. However, it remains controversial about the role of ERp29 gene in carcinogenesis and need to be clearly defined. Ye *et al.* showed that ERp29 is overexpressed in lung cancer tissues. Knockdown of ERp29 inhibited the migration of lung adenocarcinoma cells and enhanced the chemosensitivity of cells to gemcitabine [7]. Other findings indicated that ERp29 expression correlated with tumor growth rate and knockdown of ERp29 by shRNA in non-invasive MCF-7 breast cancer cells reduced tumor formation [11]. These clues suggest that ERp29 contributes to tumorigenesis. In contrast, colon cancer COLO-205 cells with high expression of ERp29 were found to grow more slowly than SW-620 cell lines. A recent study demonstrated that expression of ERp29 in MDA-MB-231 cells suppressed tumor growth in nude mice xenograft model by decreasing the cell proliferative index. Furthermore, over-expression of ERp29 could up-regulate the genes with tumor suppressive function, e.g., E-cadherin, cyclin-dependent kinase inhibitor and spleen tyrosine kinase [8]. These data support a role of ERp29 in negatively regulating cell tumorigenesis. However, the precise role of ERp29 in GC remains unclear.

In this study we used qRT-PCR and immuno-histochemical staining to detect the expression levels of ERp29 in gastric carcinoma. We further analyzed the relationship between the ERp29 expression and clinicopathological features. Finally, we used ERp29-overexpressing stable clones to examine the effects of ERp29 on proliferation and migration of GC cells and the probable molecular mechanisms. We found that ERp29 might act as an anti-oncogene in GC. ERp29 could inhibit GC cell growth and migration, and might suppress the tumorigenicity of GC by regulating the epithelial –mesenchymal transition (EMT).

## RESULTS

### ERp29 is down-regulated in gastric tumor tissues and cell lines

**Figure 1:**
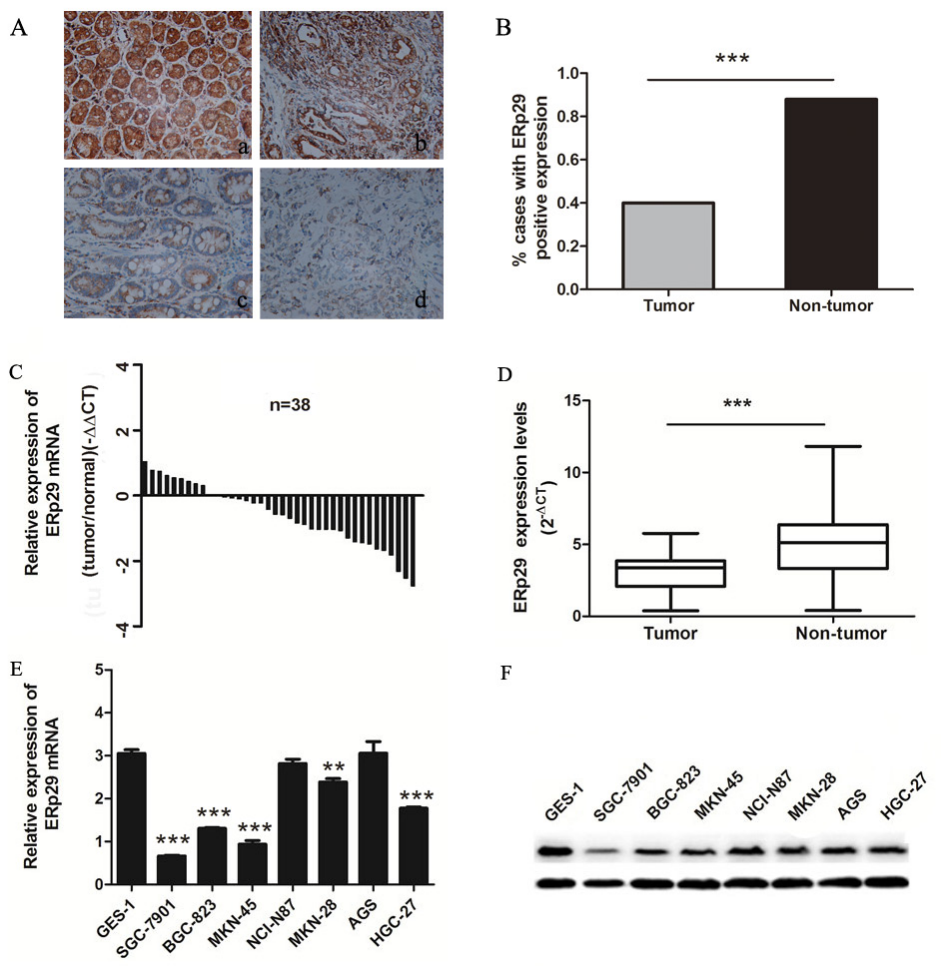
Expression of ERp29 in gastric cancer tissues and cell lines **(A)** Expression of ERp29 was performed with Immunohistochemical (IHC) staining in non-tumor gastric tissues and gastric cancer tissues. Original magnification: ×200. (a) Positive ERp29 expression in non-tumor gastric tissue. (b) Positive ERp29 expression in gastric cancer tissue. (c) Negative ERp29 expression in non-tumor gastric tissue. (d) Negative ERp29 expression in gastric cancer tissue. **(B)** Quantification of ERp29 expression by IHC analysis. **(C)** and **(D)** Elevated expression of ERp29 mRNA in 38 pairs of gastric cancer tissues. Data is shown as 2^-ΔCt^ (***P < 0.001). **(E)** and **(F)** Expression of ERp29 in human gastric cancer cell lines and normal gastric mucosal epithelial cell line. The mRNA levels of ERp29 were examined by qRT-PCR (compared to GES-1 ***P < 0.001, **P<0.01 ), and protein levels of ERp29 were examined by qRT-PCR and western blotting respectively.

To investigate the expression of ERp29 in gastric cancer tissues, we first used immunohistochemical staining to evaluate the ERp29 protein levels in GC tissue microarray sections, which was obtained from a total of 75 individuals. The results showed that ERp29 was located mainly in the cytoplasm of gastric carcinoma cells (Figure 1A). Among the 150 specimens, ERp29 staining was detected positive in 40 % (30 out of 75) of the gastric cancer compared with 88 % (66 out of 75) of adjacent non-tumor tissues, indicating that ERp29 expression in gastric carcinoma was lower than adjacent non-tumor tissues (Figure 1B, ***P<0.001). In addition, we used qRT-PCR to detect the ERp29 mRNA levels in 38 pairs of gastric cancer tissues and adjacent non-tumor tissues. The results showed that ERp29 mRNA levels were also lower in gastric cancer tissues than those in adjacent tissues (Figure 1C&1D, ***P<0.001). To further investigate the expression of ERp29 in gastric cancer cells, we used qRT-PCR and western blot analysis to examine ERp29 expression in gastric cancer cell lines and normal gastric mucosal epithelial cell line (GES-1). Compared to GES-1, the ERp29 mRNA and protein levels were lower in SGC-7901, BGC-823, MKN-45 and MKN-28 gastric cancer cell lines, indicating that the expression of ERp29 was down regulated in most gastric cancer cells (Figure 1E&1F, Supplementary Figure 1). These data suggested that ERp29 was lowly expressed in GC tissues and cells.

### ERp29 expression level is correlated with clinicopathological features and survival rate in GC patients

**Figure 2:**
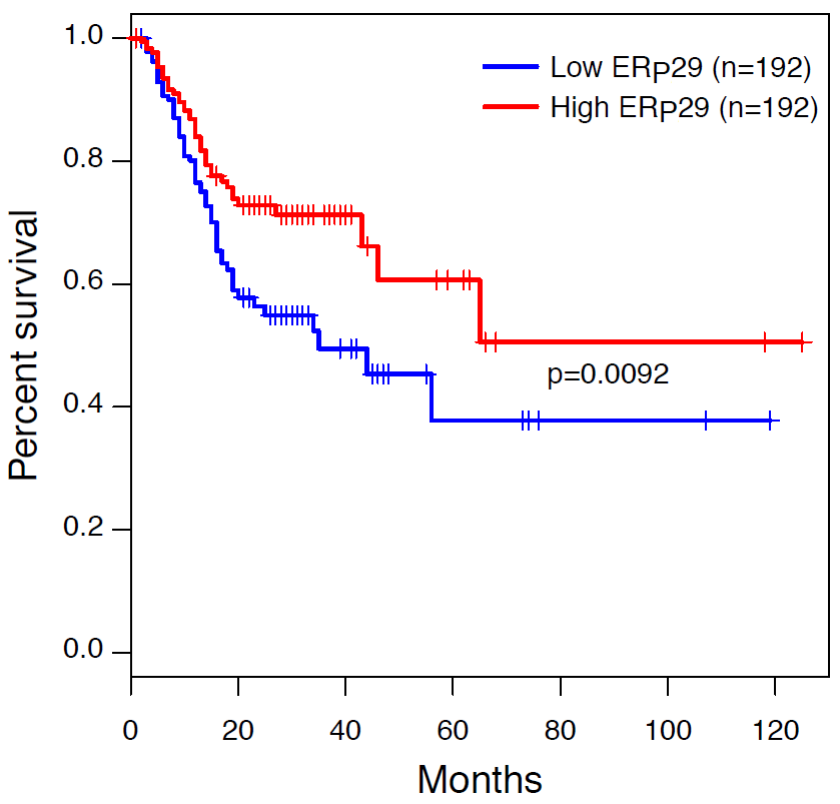
Kaplan-Meier survival curves in gastric carcinoma from The Cancer Genome Atlas (TCGA) dataset Patients with ERp29 high staining had a significantly better prognosis than those with low staining (***P < 0.001). Survival curves were explored by Kaplan–Meier method, and differences between two groups were evaluated by the log-rank test.

To elucidate the association between ERp29 mRNA expression and clinicopathological characteristics in human GC, 38 malignant tumors were analyzed. The clinical and pathologic characteristics of 38 GC patients were showed in Table 1. Cases were classified according to the ERp29 expression into two groups: ERp29 low expression (n = 28) and ERp29 high expression (n = 10). Chi-square test suggested that 13.04% (3/23) of ERp29 high expression were observed in GC tissues with diameter more than 5cm whereas 46.67% (7/15) in diameter less than 5cm. In addition, the ERp29 high expression rate was 50% (7/14) in TNM Stage I+II and 12.5% (3/24) in TNM Stage III+IV. These results indicated that ERp29 high expression levels in GC tissues were significantly correlated with smaller tumor size (*P=0.03) and earlier TNM stage (*P=0.02). However, there were no significant relationships between the ERp29 expression and other clinicopathologic features such as age (*P*=0.726), gender (*P*=1) or Lauren’s classification (*P*=0.719). In order to investigate the association between ERp29 expression and survival of GC patients, we obtained the Kaplan-Meier survival curve from The Cancer Genome Atlas (TCGA) dataset, the results of which showed that the survival rate of the cases with ERp29 high expression was significantly longer than those with low expression (Figure 2, |***P<0.001).

**Table 1:** Clinicopathologic associations of ERp29 mRNA in 38 pairs of gastric cancer and paired non-cancer tissues

Clinicopathological variable	No. of patients	ERp29 staining		P value
		Weak	Strong	
Age (years)				0.726
<60	23	16	5	
≥60	15	12	5	
Gender				1
Male	23	16	6	
Female	15	12	4	
Tumor size (cm)				0.03*
≤5	15	8	7	
>5	23	20	3	
Lauren’s classification				0.719
Intestinal-type	20	14	6	
Diffuse-type	18	14	4	
TNM stage				0.02*
I+II	14	7	7	
III+IV	24	21	3	

### ERp29 inhibits growth of gastric cancer cells *in vitro*

**Figure 3:**
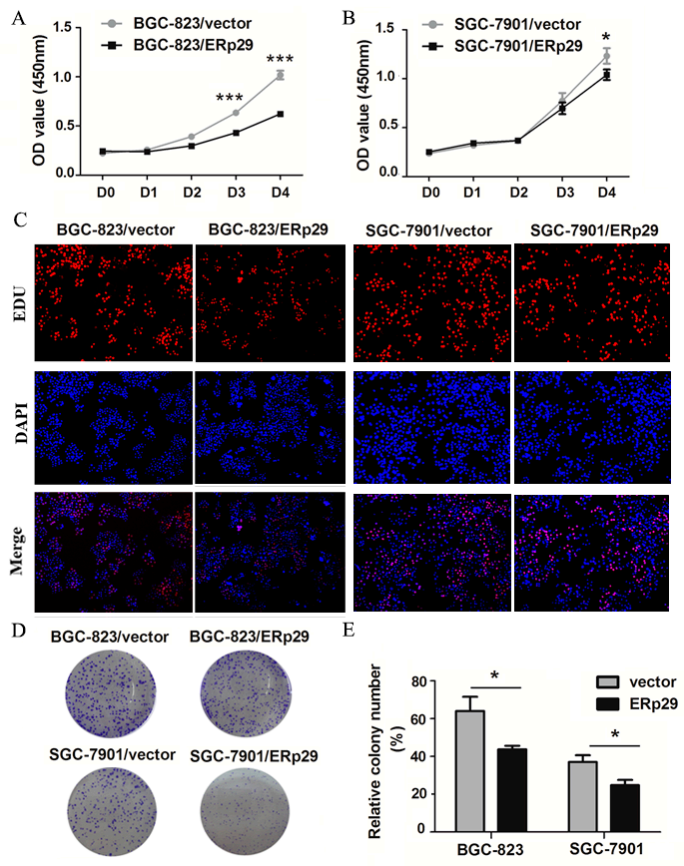
Effects of ERp29 on cell growth in GC cells **(A)** and **(B)** Effects of ERp29 overexpression on GC growth using CCK8 assay. (*P < 0.05, ***P < 0.001). Cells were seeded in 96-well plates for 4 days, and cell viability was assessed at indicated times. **(C)** Effects of ERp29 overexpression on GC growth using the EDU assay. **(D)** and **(E)** Effects of ERp29 overexpression on GC growth using the plate colony formation assay. Cells were seeded in 96-well plates for 14 days, and cell colonies were stained and counted. The data represents mean ± SD of three independent experiments (*P < 0.05).

ERp29 expression was negatively related to tumor size in GC tissues. Therefore, we examined whether overexpression of ERp29 in GC cells affected cell growth. We chose the ERp29 low-expression cell lines BGC-823 and SGC-7901 for further studies. ERp29-expressing lentivirus vector was transfected into BGC-823 and SGC-7901 cells to generate ERp29 overexpression models (BGC-823/ERp29; SGC-7901/ERp29). CCK8 assay was used to evaluate GC cell proliferation. As shown in Figure 3A&3B, compared with the control group, overexpression of ERp29 could inhibit the proliferation of BGC-823 and SGC-7901. The difference was statistically significant at day 3. However, up-regulation of ERp29 had no effect on proliferation of GES-1 (Supplementary Figure 2). To further characterize the effect of ERp29 on cell growth, we performed EDU assay and colony formation assay. EDU staining results showed a lower rate of EDU positive cells in BGC-823/ERp29 and SGC-7901/ERp29 cells than in control cells (Figure 3C). Colony formation assay revealed that ERp29 overexpression cells formed fewer colonies than control cells (Figure 3D&3E, *P<0.05). Thus, together, these results implicated that ERp29 inhibits human GC cell growth and proliferation *in vitro*.

### ERp29 suppresses migration of gastric cancer cells *in vitro*

**Figure 4:**
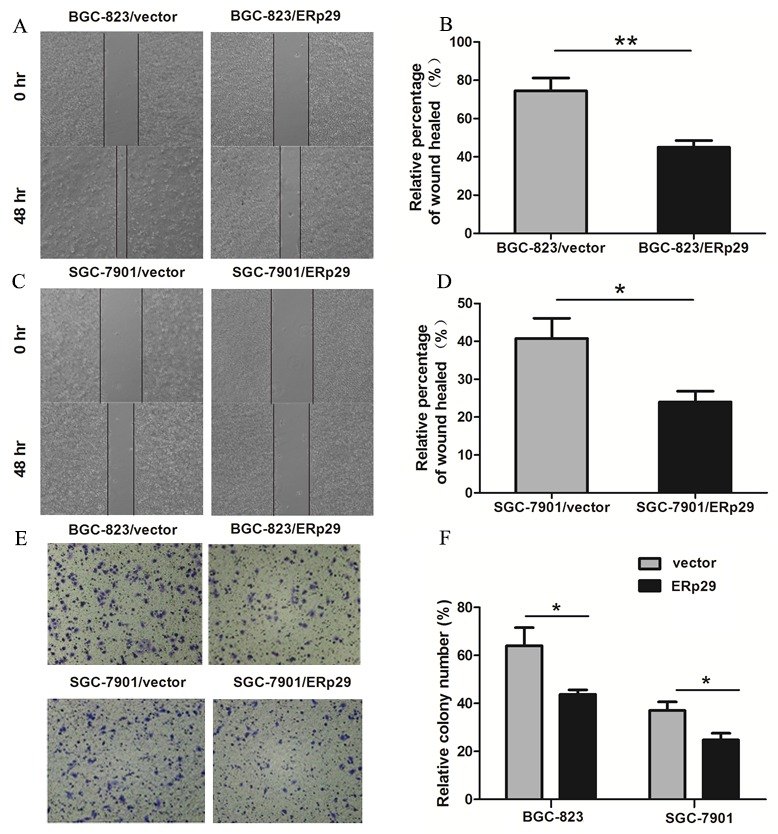
Effects of ERp29 on cell migration in GC cells **(A)** and **(B)** Representative images of scratch wounds in BGC-823/vector and BGC-823/ERp29 cells. Cells were seeded in 6-well plates for 48h, and the relative distances of cell wounds were measured at 0h and 48h (**P<0.01). **(C)** and **(D)** Representative images of scratch wounds in SGC-7901/vector and SGC-7901/ERp29 cells (*P<0.05). **(E)** Representative photographs of migrated ERp29 overexpression cells through chambers’ membrane. **(F)** The average number of migrated ERp29 overexpression cells. Cell number was counted from five randomly-selected microscopic fields (*P <0.05). The data is shown as mean ± SD of three independent experiments.

ERp29 expression was negatively correlated with TNM stage in GC tissues, suggesting that ERp29 may be involved in the metastasis of gastric cancer cells. Wound healing assay was used to examine migration ability of GC cells. BGC-823/vector cells nearly closed the wound 48 h after scratching, whereas BGC-823/ ERp29 cells failed to heal the wound (Figure 4A&4B). The results were observed in the SGC-7901 cell lines (Figure 4C&4D). To further detect the effect of ERp29 on cell migration, we performed transwell migration assays. The number of cells migrating through the chamber in BGC-823/ERp29 was 52±14.35 which was lower than in control group (94.75±10.21) (Figure 4E&4F, *P<0.05). The same result was also observed in SGC-7901 cell lines with SGC-7901/ERp29 (30±10.1) and SGC-7901/vector (61.95±21.95) (Figure 4E&4F, *P<0.05). These data indicated that ERp29 inhibits human GC cell migration *in vitro*.

### ERp29 suppresses tumorigenicity of gastric cancer cells in nude mice *in vivo*

**Figure 5:**
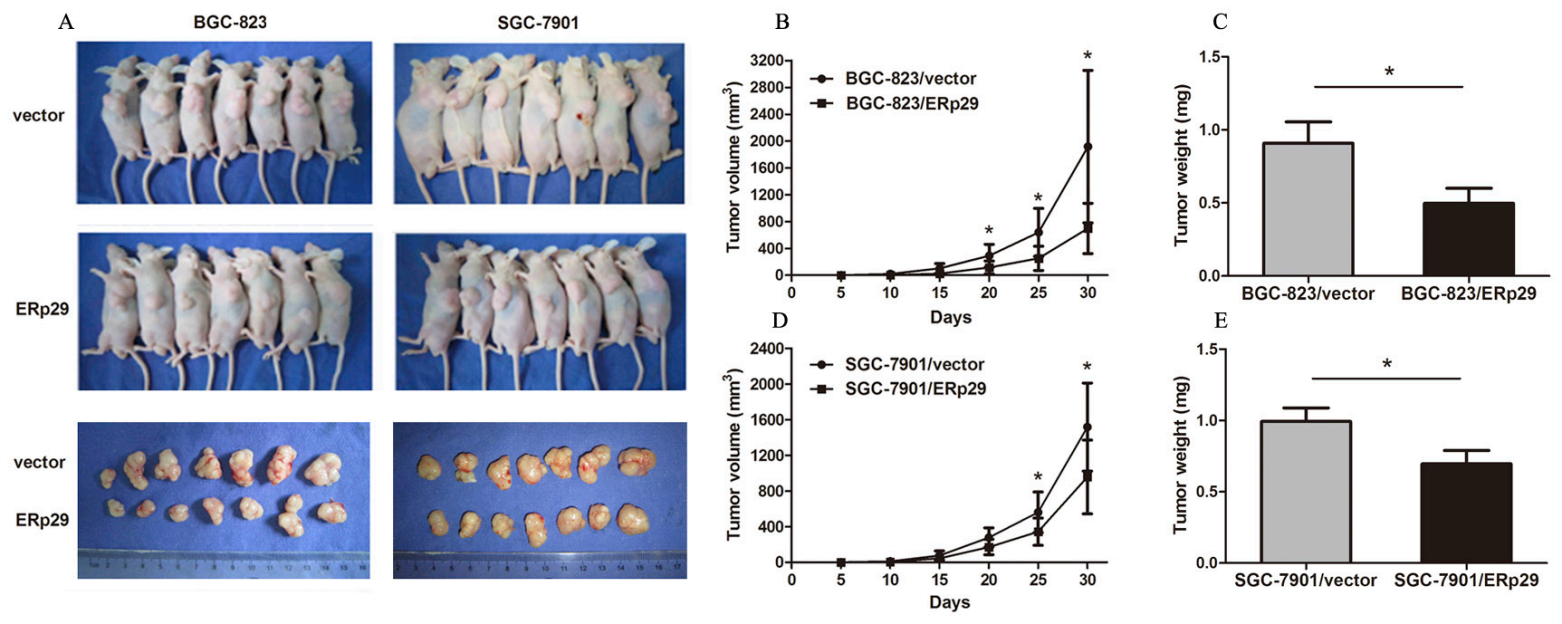
Effects of ERp29 on tumor growth *in vivo* **(A)** Photographs of tumors derived from BGC-823/vector, BGC-823/ERp29, SGC-7901/ vector and SGC-7901/ ERp29 cells in nude mice. **(B)** and **(D)** Growth kinetics of tumors in nude mice. Tumor diameters were measured every 5 days during the 30 days. **(C)** and **(E)** Average weights of tumors in nude mice. Data are expressed as the mean ± SD.

In order to clarify the *in vivo* tumorigenicity function of ERp29, GC cells were injected subcutaneously into the nude mice and tumor formation was monitored. On day 30, mice were sacrificed under anesthesia. Tumor weights were measured and the inhibition rates of tumor growth were calculated. Tumors grew slower in BGC-823/ERp29 and SGC-7901/ERp29 groups compared to the groups of BGC-823/vector and SGC-7901/vector, respectively (Figure 5A&5B&5D). Furthermore, tumor weights were lower in BGC-823/ERp29 group than that in the group of BGC-823/vector (0.50±0.25g vs. 0.91±0.36g, *P<0.05, Figure 5C). The same result was also observed in SGC-7901 groups (SGC-7901/ERp29 0.70±0.22g vs SGC-7901/vector 0.99±0.36g, *P<0.05, Figure 5E). These findings suggested that ERp29 could inhibit the cell growth *in vivo*.

### ERp29 inhibits EMT process in gastric cancer cells

**Figure 6:**
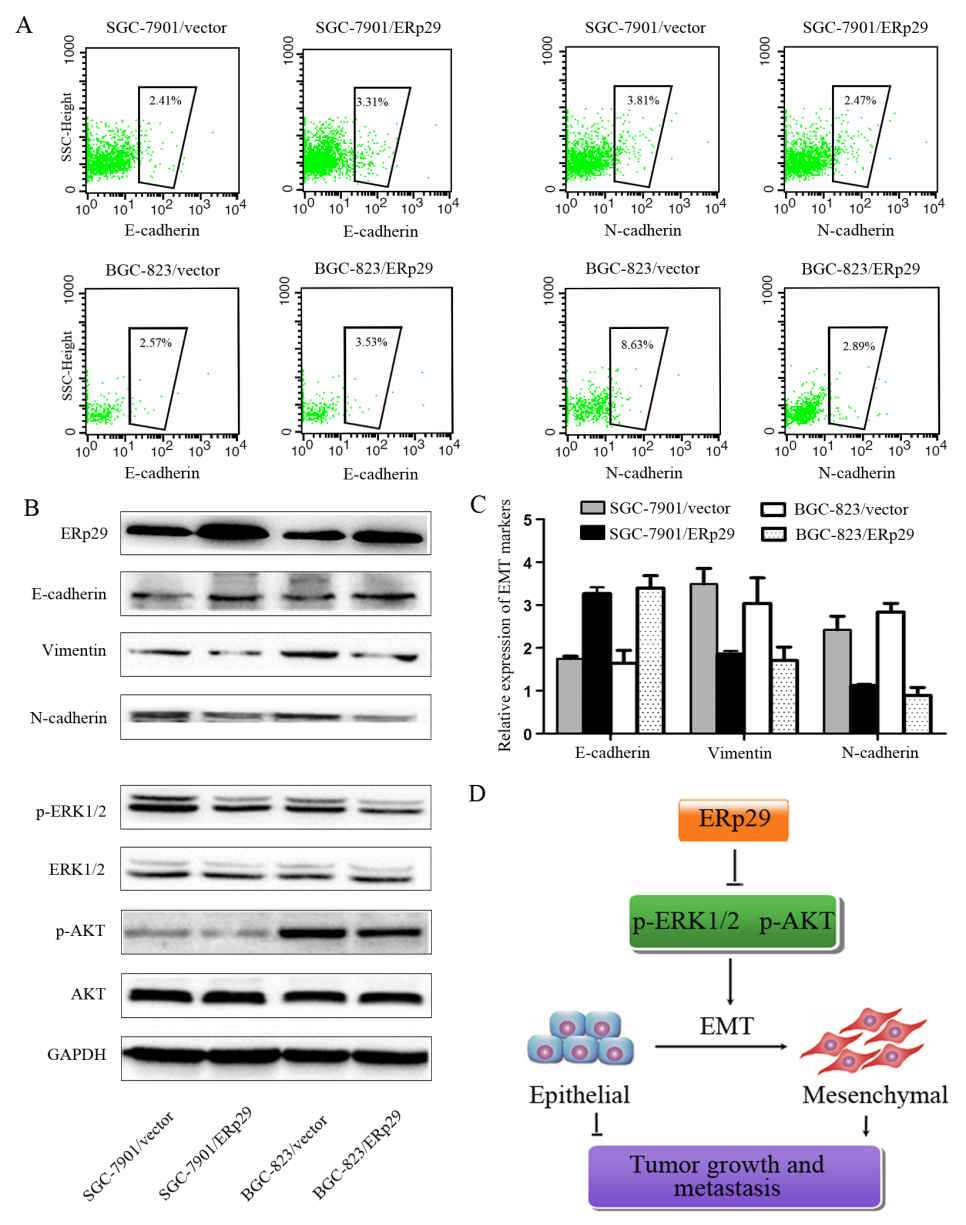
Effects of ERp29 on EMT process of GC cells **(A)** Flow cytometry analysis of E-cadherin and N-cadherin expression on SGC-7901/vector, SGC-7901/ ERp29 BGC-823/vector and BGC-823/ERp29 cells. The number of cadherin positive cells inside the black box were represented by a percentage value. **(B)** The protein levels of EMT markers, ERK1/2 and AKT phosphorylation levels were assayed by western blot analysis. GAPDH was used as a loading control. **(C)** qRT-PCR analysis of E-cadherin, vimentin and N-cadherin expression on SGC-7901/vector, SGC-7901/ ERp29 BGC-823/vector and BGC-823/ERp29 cells. **(D)** A hypothetical schematic of the regulation of ERp29 on EMT in GC cells.

Studies have shown that ERp29 may act as a tumor suppressor gene in breast cancer by regulating EMT process. We thus investigated whether ERp29 could affect EMT in GC cells. The efficacy of ERp29 overexpression is shown in Figure 6B. The levels of ERp29 protein were enhanced in BGC-823/ERp29 and SGC-7901/ERp29 cells in comparison to their controls. As expected, Overexpression of ERp29 leads to increased expression of E-cadherin and reduced expression of mesenchymal markers N-cadherin and Vimentin, suggesting that ERp29 may inhibit EMT process (Figure 6A&6B&6C, Supplementary Figure 3A). ERK and AKT signaling have been reported to be involved in EMT. We thus sought to assess whether ERp29 mediates its effects via the ERK and AKT signaling. The results revealed that the phosphorylation of ERK1/2 and AKT were down-regulated in ERp29 overexpression cells (Figure 6B, Supplementary Figure 3B). These findings suggest that ERp29 may stimulate the inactivation of ERK1/2 and AKT, contributing to inhibit EMT process and then reduce the tumorigenicity of human gastric cancer cells (Figure 6D).

## DISCUSSION

ERp29, a novel 29-kDa endoplasmic reticulum (ER) protein, belongs to the protein disulfide isomerize (PDI) family and has been reported to be closely associated with the tumor development and progression [9, 12]. However, the expression and function of ERp29 in gastric cancer remain largely unknown. Recently, abnormal regulation of other PDI family proteins such as ERp57, AGR2 and TXNDC5 has been found in GC. Leys *et al.* suggested that ERp57 expression is down-regulated in GC and associated with tumor invasion depth, TNM stage, and patient survival [13]. Tsuji *et al.* showed that extracellular AGR2 can activate stromal fibroblasts and plays important roles in the progression of gastric signet-ring cell carcinoma (SRCC) [14]. Zhang *et al.* suggested that TXNDC5 was overexpressed in GC and could promote the growth and invasion of gastric cancer cells [15].

In the present study, we found that ERp29 expression is substantially down-regulated in 38 GC tissues compared with adjacent non-tumor tissues by qRT-PCR, indicating that ERp29 may suppress tumorigenicity in GC. We further analyzed the pathologic characteristics of the 38 GC cases and found that tumor with high ERp29 expression had inclinations towards smaller tumor size and earlier TNM stage. In addition, Kaplan-Meier analysis showed that low expression of ERp29 was closely correlated to the short-term survival and prognosis of patients with gastric cancer. These data indicated that ERp29 may be associated with tumor cell proliferation and metastasis in gastric cancer.

To date, the biological function of ERp29 in gastric cancer has not been definitively reported. In this study, we used the lentivirus infection method to up-regulate ERp29 in two gastric carcinoma cell lines. The CCK8, EDU and clonogenic assays suggested that overexpression of ERp29 inhibits proliferation and colony formation of GC. The wound-healing and transwell assays indicated that ERp29 overexpression significantly impaired the migration ability of GC cells. *in vivo* result confirmed that overexpression of ERp29 could inhibit the growth of subcutaneous tumor in nude mice. Thus, all the above suggest that ERp29 may act as a novel tumor suppressive factor involved in the formation or progression of gastric cancer.

Cancer progression from early stage carcinomas to invasive malignancies is associated with the loss of epithelial differentiation and gain of mesenchymal phenotype, which is accompanied by increased cell motility and invasion [16-18]. Emerging evidences suggested that the epithelial-mesenchymal transition (EMT) is a key GC progression driver and plays an important step in GC invasion, metastasis and relapse [19]. EGFL7 promotes infiltration and metastasis of GC by activating EMT [20]. The over-expression of FoxM1 promotes cell migration, invasion and proliferation by up-regulating vimentin while down-regulating E-cadherin in GC cells [21]. The knockdown of ZEB2 could inhibit invasion and migration of GC cells, along with the up-regulation of E-cadherin and down-regulation of mesenchymal cell markers fibronectin and vimentin [22]. Recently, it has been reported that ERp29 negatively regulates the EMT process in MDA-MB-231breast cancer cells. In this study, we found that overexpression of ERp29 could increase the epithelial marker E-cadherin and decrease the mesenchymal cell markers N-cadherin and Vimentin, implying that ERp29 overexpression may suppress EMT in gastric cancer.

Previous reports show that activation of MEK/ERK and PI3K/AKT contributes to growth, invasion, and EMT in human cancers [23-26]. Those two pathways are involved in EMT by regulating the transcription factors, Twist, Slug and Ets1 [8, 27, 28]. Mechanistic studies have demonstrated that AKT activation inhibits the expression of E-cadherin by up-regulating snail activity [29]. Chang *et al.* found that activation of PI3K/AKT signaling pathway can promote the expression of mesenchymal phenotype and inhibit the apoptosis of prostate cancer cells [30]. In addition, MEK/ERK signaling pathway was found to be involved in the EMT process induced by HOXB7 and TGF-β [31, 32]. In this study, we showed that over-expression of ERp29 in GC cells results in the inactivation of ERK1/2 and AKT phosphorylation, causes mesenchymal–epithelial transition (MET) and suppresses tumor progression.

Though further investigations are needed to identify the possible roles and mechanisms of ERp29, our study is the first to evaluate its clinical significance in GC. Our study also has some limitations, including a low number of the study sample and the fact that no attempt was made to measure the transcription factors of EMT. The other limitations are the lack of data about gene knockdown experiments.

In conclusion, our studies indicate that ERp29 is down-regulated expression in gastric cancer tissues, and could inhibit GC cell proliferation and migration *in vitro* and *in vivo*. In addition, further mechanistic studies showed that ERp29 inhibits GC cell EMT by regulating MEK/ERK and PI3K/AKT pathways. Thus, we accept our hypothesis that ERp29 inhibits tumorigenicity by suppressing EMT in gastric cancer. We believe that these findings are helpful to understand the tumor suppressive role of ERp29 in GC and underscore the possibility that ERp29 may serve as a potential anticancer drug target for treatment of GC.

## MATERIALS AND METHODS

### Patient samples

38 GC patients who underwent radical resection were recruited randomly between 2012 and 2014 at the Department of Surgery, Ruijin Hospital, Shanghai, China. All samples were confirmed by pathological diagnosis. The corresponding non-tumor gastric tissue was obtained at least 6 cm from the tumor. All tissue samples were frozen in liquid nitrogen immediately after the resection and stored at -80°C until RNA extraction. The study was approved by the Shanghai Jiao Tong University Medical School institutional review board and written informed consent was obtained from all participants.

### Cell lines and cell culture

Gastric cancer cell lines BGC-823, MKN-45, AGS, SGC-7901, MKN-28, NCI-N87, HGC-27 and immortalized gastric epithelial cell line GES-1, were cultured at 37°C in a humidified incubator with 5% CO2 in RPMI 1640 medium supplemented with 10% FBS (Gibco).

### Plasmids construction and transfection

ERp29 cDNA ORF was cloned into the pLVX-AcGFP-N1-ERP29 for lentivirus production. The lentivirus-packing plasmid was transfected into gastric cell lines and the stable transfections were screened and cultured by purimycine. Finally the stable cell lines were identified by western blotting.

### Tissue microarray and immunohistological analysis

Gastric cancer tissue arrays were purchased from the National Engineering Center for BioChips in Shanghai, China. Monoclonal anti-ERp29 was used at a dilution of 1: 300 (Abcam). The slides were evaluated by a single board-certified pathologist (RRT). Immunohistochemistry (IHC) scores were calculated by multiplying the percentage of positive cells (0 is <5% positive cells, 1 is 5%-25% positive cells, 2 is 25%-50% positive cells, 3 is 50%-75% positive cells and 4 is > 75% positive cells) by stain intensity (0 is no staining, 1 is weak staining, 2 is moderate staining and 3 is strong staining) in five different high power fields for each section. The IHC score of 0-4 was defined as weak expression and 4-12 as strong expression.

### Quantitative realtime-PCR (qRT-PCR)

Total RNA was extracted using Trizol Reagent kit (Invitrogen) and cDNA was synthesized with oligo (dT) primers by using the Reverse Transcription kit (Takara). PCR amplification was performed using SYBR Green PCR master mix kit (Qiagen). Primers used for qRT-PCR analysis of ERp29 expression are 5’-CCTGGATACGGTCACTTTCTACA-3’ (forward) and 5’-AGTTTTCAGCAAGACGCTTGA-3’ (reverse). Primers used for analysis of the internal control reference GAPDH are 5’-TTGGCATCGTTGAGGGTCT-3’ (forward) and 5’-CAGTGGGAACACGGAAAGC-3’ (reverse). The comparative threshold cycle (Ct) method was used to determine the relative level of gene expression.

### Cell proliferation assays

CCK8 assay: Cells were cultured in a 96-well plate at a concentration of 1×10^4^ cells/ml and incubated for 4 days; OD450 was measured 2 h after adding CCK-8 at 0, 24, 48, 72 and 96 h.

EdU assay: Cells were seeded onto 96-well plates at a final density of 1×10^4^ cells/ml and incubated for 24 hours. Cell proliferation was then measured by Cell-Light™ EdU fluorescence microscopy detection kit (RiboBio).

Plate colony formation assay: Cells were resuspended in RPMI 1640 containing 10% FBS and layered onto 6-well plates (5×10^2^ cells/well). The cells were incubated for 2-3 weeks, formalin-fixed and stained with 0.4% crystal violet. Colonies containing 50 cells or more were counted.

### Cell migration and wound healing assays

Cell migration was analyzed using a transwell chamber assay (Corning). Cells (2.0×10^5^) in 200 μl of serum-free medium were added to the top chamber while 600ul of medium containing 10% FBS was added to the lower chamber. Cells that migrated into the lower chambers were fixed in 4% paraformaldehyde, stained with 1% crystal violet after 24 hours and counted in six random fields. For the wound healing assay, 5×10^5^ cells were seeded in 6-well plates and cultured for 24h. The cell monolayers were wounded with a pipette tip. The wound closure was observed every 24 h.

### Western blot analysis

Cell lysates were harvested using RIPA cell lysis buffer. An equal amount (15 ug) of total cellular protein was separated by 12.5% SDS-PAGE and transferred to polyvinylidene difluoride (PVDF) membranes (Millipore). The membranes were then blocked with 5% non-fat milk for 2 h and incubated overnight with primary antibodies against ERp29 (Abcam), Akt, p-Akt, ERK, p-ERK(Cell Signaling Biotechnology), E-cadherin, vimentin, N-cadherin (Santa Cruz). After incubated with HRP conjugated-secondary antibody for 2 h at room temperature, the proteins were visualized by enhanced chemiluminescence (ECL).

### Flow cytometry

Cells (1.0×10^6^) were incubated with primary antibodies (0.5ug) against E-cadherin and N-cadherin (Abcam) at room temperature for one hour. Then the cells were washed with PBS for three times, incubated with fluorescent secondary antibodies (1:100) for 30 minutes and finally followed by FACS analysis.

### *In vivo* tumorigenesis

SPF grade male BALB/c nude mice were housed at a specific pathogen-free environment in the Animal Laboratory Unit, School of Medicine, Shanghai Jiao Tong University, China. Cells (2.0×10^6^) in 200 μl of RPMI 1640 were subcutaneously injected into 4-week-old male nude mice. The diameters of each tumor including the length (L) and width (W) were measured every 5 days with calipers and the volume was calculated using the formula: (W+L)/2×W×L×0.5236.

### Statistical analysis

Statistical analysis was performed using SPSS 16.0 software. Data were presented as the mean±standard deviation (SD). The correlation between ERp29 expression levels and clinicopathologic parameters were analyzed by the Pearson χ2 test. Differences between groups were calculated by using the Student’s t-test or one-way ANOVA. *P* < 0.05 was considered to be statistically significant.

## SUPPLEMENTARY MATERIALS FIGURES


