# Supplementary material for: ERp29 inhibits tumorigenicity by suppressing epithelial mesenchymal transition in gastric cancer
Source: Oncotarget. 2017 Aug 12;8(45):78757–66. doi: 10.18632/oncotarget.20225 (PMC5667996; doi:10.18632/oncotarget.20225)
Supplement: Supplementary file 1 [file oncotarget-08-78757-s001.pdf]

ERp29 inhibits tumorigenicity by suppressing epithelial mesenchymal transition in gastric cancer

SUPPLEMENTARY MATERIALS

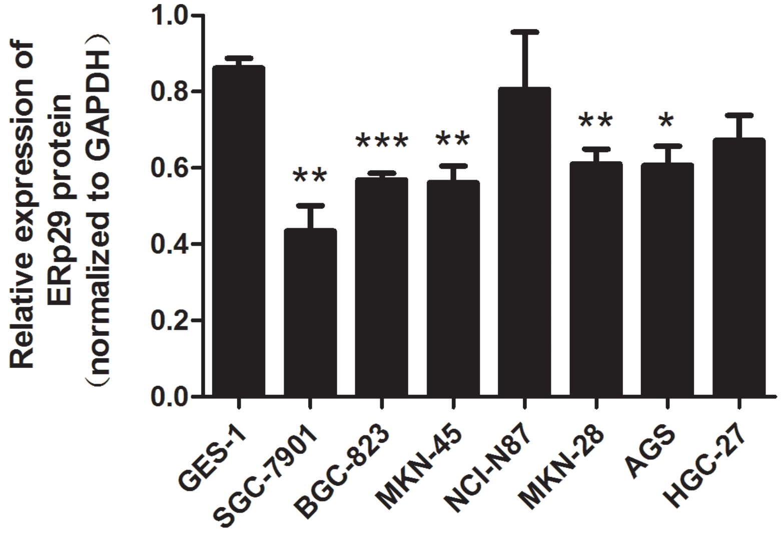

Supplementary Figure 1: Relative expression of ERp29 protein in human gastric cancer cell lines and normal gastric mucosal epithelial cell line. (Compared to GES-1 ***P < 0.001, **P<0.01, *P < 0.05).

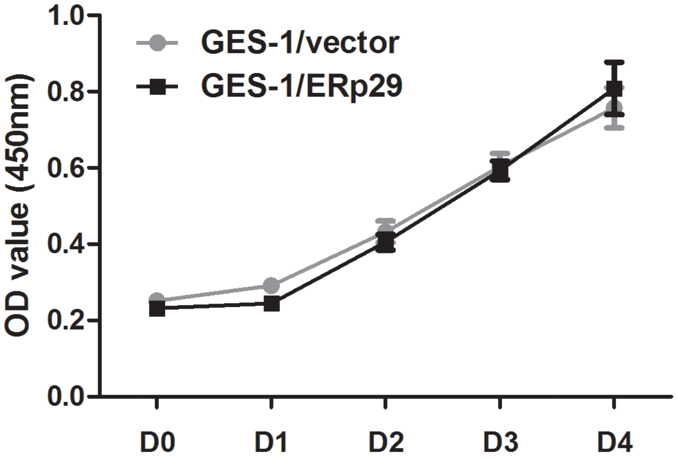

Supplementary Figure 2: Effects of ERp29 overexpression on cell growth in GES-1.

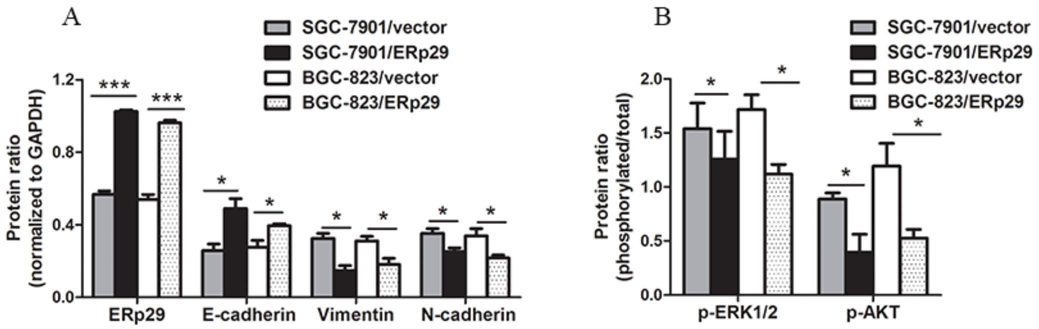

Supplementary Figure 3: Protein ratio of EMT markers, ERK1/2 and AKT phosphorylation levels. **(A)** Protein ratio of EMT markers in SGC-7901 and BGC-823 cells (*P<0.05). **(B)** Protein ratio of ERK1/2 and AKT phosphorylation levels in SGC-7901 and BGC-823 cells (*P<0.05).
